# *Salmonella* Infection in Chronic Inflammation and Gastrointestinal Cancer

**DOI:** 10.3390/diseases7010028

**Published:** 2019-03-10

**Authors:** Lang Zha, Shari Garrett, Jun Sun

**Affiliations:** Division of Gastroenterology and Hepatology, Medicine, University of Illinois at Chicago, Chicago, IL 60612, USA; lzha3@uic.edu (L.Z.); sgarre5@uic.edu (S.G.)

**Keywords:** cancer, dysbiosis, inflammation, infection, *Salmonella*, microbiome, Wnt/β-catenin

## Abstract

*Salmonella* not only causes acute infections, but can also cause patients to become chronic “asymptomatic” carriers. *Salmonella* has been verified as a pathogenic factor that contributes to chronic inflammation and carcinogenesis. This review summarizes the acute and chronic *Salmonella* infection and describes the current research progress of *Salmonella* infection contributing to inflammatory bowel disease and cancer. Furthermore, this review explores the underlying biological mechanism of the host signaling pathways manipulated by *Salmonella* effector molecules. Using experimental animal models, researchers have shown that *Salmonella* infection is related to host biological processes, such as host cell transformation, stem cell maintenance, and changes of the gut microbiota (dysbiosis). Finally, this review discusses the current challenges and future directions in studying *Salmonella* infection and its association with human diseases.

## 1. Introduction

*Salmonella* is a Gram-negative bacterial genus of the Enterobacteriaceae family with a strong pathogenicity that can cause cross-infection between humans and animals. In the United States, approximately 1.4 million people are infected with *Salmonella* each year [1]. *Salmonella* causes fever, diarrhea, gastroenteritis, and sepsis in humans, as well as intestinal damage in both humans and animals. In addition, *Salmonella* infections are reported to increase the risk of immune-related diseases such as arthritis [2,3] and inflammatory bowel disease (IBD) [4]. Furthermore, chronic infection of *Salmonella* may lead to gallbladder and colorectal cancer [5,6].

We performed an electronic literature search of papers written in English in the MEDLINE database via PubMed. Searches included combinations of the following terms: *Salmonella*, infection, microbiome/microbiota, inflammatory bowel disease, inflammation, cancer, nuclear factor kappa B, and stem cells. Papers without clear relevance to the role of *Salmonella* infection, inflammation or cancer were excluded.

Here, we review the acute and chronic *Salmonella* infection. We summarize the current research progress of *Salmonella* infection and its contribution to inflammation and cancer. We also discuss the potential mechanisms of *Salmonella* infection and its association with various human diseases.

## 2. Clinic and Epidemiologic Facts of *Salmonella* Infection

Foodborne diseases cause 1 in 10 persons to fall ill each year and one of every four diarrheal patients has been infected with *Salmonella* [7]. In the United States, *Salmonella* is the second most prevalent foodborne infection [8]. In Europe, there were about 94,625 *Salmonella* infections in 2015 [9]. The preference of “ready-to-eat”, raw or lightly cooked foods is a potential reason for the high number of infections in Western countries. Approximately 2600 serotypes have been described based on the phenotypic identification of the somatic and flagellar antigens of *Salmonella* [10].

Of the 2600 identified *Salmonella* serotypes, many cause a range of symptoms in different hosts. Based on its virulence factors, *Salmonella* can be divided into typhoidal and non-typhoidal *Salmonella* (NTS) serovars. *Salmonella enterica* (*S. enterica*) serotypes Typhi and Paratyphoid typically cause typhoid in humans, which is characterized by systemic infection, fever and gastrointestinal symptoms like diarrhea. In contrast, *S. enterica* serotype Typhimurium (*S.* Typhimurium) typically presents as enterocolitis in humans and cattle and is known to cause systemic infection in mice [11].

Typhoidal *Salmonella* infection typically occurs in under-developed countries due to poor sanitation practices [12], but NTS is prevalent worldwide [12,13]. In fact, there are different clinical manifestations between the typhoidal *Salmonella* and NTS infection. The manifestations of NTS infection are acute clinical symptoms, such as diarrhea, fever, abdominal pain, nausea, and vomiting. The symptoms are usually self-limiting as the fever usually returns to normal in 72 h and diarrheal symptoms disappear in 3–7 days [14]. Typhoidal *Salmonella* infection can cause systemic symptoms without the manifestation of intestinal symptoms. Systemic symptoms include a gradual onset of sustained fever, hepatosplenomegaly, and rash [15]. Because antibiotic therapy prolongs fecal excretion of *Salmonella* [16], it is not recommended to treat an NTS-infected patient with antibiotics unless the patient is immunocompromised. For typhoidal *Salmonella-*infected patients, fluoroquinolones are usually the first choice to treat the bacteria, and third generation cephalosporins can be an alternative treatment when typhoidal *Salmonella* is resistant to fluoroquinolones [17]. The acute manifestations are easy to identify and treat by patients and physicians but chronic asymptomatic infection may produce more serious problems, such as IBD and cancer. Thus, typhoidal *Salmonella* infections can cause patients to become carriers who may not only develop more severe diseases but can spread the pathogen to healthy individuals.

## 3. Acute *Salmonella* Infection

*Salmonella* infections begin with ingestion of the organism from contaminated food or water. Once ingested, *Salmonella* must overcome the acidity of the stomach. *Salmonella* can develop an adaptive acid-tolerance response when exposed to gastric pH, promoting its survivability and gastric invasiveness [18,19]. In the intestine, *Salmonella* can invade the epithelium through three distinct routes: by adhering and entering into M-cells or epithelial cells, or by infecting mononuclear phagocytes that sample the gut lumen [20].

There are many virulence factors that are involved in infection. Bacteria surface moieties, toxins, and effector proteins all play roles in invasion. Most importantly, these components can modify essential host cell-signaling pathways related to both acute and chronic infections [21,22,23]. *Salmonella*’s ability to invade host cells is regulated by *Salmonella* pathogenicity islands (SPI), which are clusters of genes that are responsible for specific virulence phenotypes and are acquired through horizontal gene transfer. SPI-1 and SPI-2 code for variants of the type 3 secretion system (T3SS) [24]. The T3SS acts as a ‘molecular syringe’ comprised of two ring-like structures at its base that span the bacterial inner and outer membrane and a needle-like structure protruding on the outside of the bacteria [25,26]. The T3SS is primarily responsible for secretion of bacterial effector proteins needed for the invasion of intestinal epithelial cells that result in intestinal secretory and inflammatory responses as well as translocating proteins for intracellular survival, replication and the establishment of systemic infection beyond the intestinal niche [27].

When bacteria interacts with the host cell, a signal is initiated inside the bacterium causing the translocators SipB and SipC to form a core complex on the host cell’s plasma membrane [21]. Effector proteins SipA and SipC will directly interact with host-cell actin, inducing cytoskeletal re-arrangements [28]. The effectors SopE, SopE2, and SopB mediate this process by activating Rho family GTPases resulting in the formation of highly organized actin structures that cause the host cell membrane to ruffle around and engulf the bacterium into a phagosome or *Salmonella*-containing vacuole (SCV) [29]. Once engulfed, the membrane shuffling is terminated by the bacterial effector protein SptP, causing the actin cytoskeleton to return to its original state [30]. SopB also activates AKT for efficient formation of the SCV and excludes Rab proteins from the SCV to delay lysosomal degradation and epithelial cell apoptosis [31,32]. SopB, SipA, and SopE promote acute intestinal inflammation and fluid secretion by disrupting tight junctions between epithelial cells and challenging the inositol phosphate signaling pathways that prevent adequate chloride secretion [21,33].

AvrA is an interesting *Salmonella* protein that plays a unique role compared to other effector molecules. We have demonstrated that AvrA inhibits the NF-κB signaling pathway and activates β–catenin signaling [34,35,36,37]. These host-bacterial interactions lead to suppression of innate immunity, inflammation, and apoptosis during infection [21,37,38]. Infection with *Salmonella-*expressing AvrA has been shown to promote intestinal permeability [39,40], activate the Wnt/β–catenin pathway, and subsequently increase proliferation [41,42,43]. In fact, AvrA plays a role in a few other pathways including mTOR, oxidative phosphorylation, and MAPK [44]. AvrA also modulates the *Salmonella* survival niche within macrophages by preventing apoptosis and bacterial propagation [45]. Bacterial effectors like AvrA provide opportunities for pathogens to “make peace” with the host and survive for extensive periods of time within host cells.

## 4. Persistent/Chronic *Salmonella* Infection in Mice and Men

Two to five percent of patients with acute *Salmonella* typhoid infections fail to completely clear the bacteria from the body, leading to chronic infection [46]. The course of chronic *Salmonella* infection has three distinct periods. A period of convalescence when carriers shed the bacteria for 3 weeks to 2 months post-infection; a period of temperance in which carriers sheds the bacteria for 3–12 months; and finally the chronic period where the organism is shed for more than a year [47]. In chronic infections, *Salmonella* can also use the gallbladder as a niche. It can invade the gallbladder directly from the liver or invade via the biliary tract directly from the small intestine or from system infection. [48]. It is presumed that once settled in the gallbladder, gallstone biofilms of *Salmonella* lead to reinfection of the intestine with subsequent transmission to new hosts via fecal shedding [49].

Studies have shown that IL-22 and IFN-γ-mediated immunity is vital for *Salmonella* eradication from cellular reservoirs [50]. An ineffective immune response or microbiota disruption can be implicated in the host’s inability to clear the organism [50]. Once persistence has been established, it is commonly detected by high anti-Vi titers [48]. The Vi antigen of *Salmonella* is coded by SPI-7 and plays a role in evading infection by masking the detection of LPS by TLR4 [50]. Therefore, the human carrier state may be associated with impaired immune function.

The most common in vivo method to study *Salmonella* infection is to use mouse models. *S.* Typhimurium instead of *S.* Typhi is commonly used in mouse models because the disease phenotype is similar to human typhoid as well as the fact that mice are unable to be infected by *S.* Typhi [51]. However, in mice, *S.* Typhimurium mimics the acute phase of typhoid fever. Several chronically infected mice models have been studied. We created a mouse model with persistent *Salmonella* infection lasting 27 weeks and investigated the physiological effects as well as the role of AvrA. We found that AvrA suppressed intestinal inflammation and inhibited the secretion of cytokines IL-12, IFN-γ, and TNF-α. AvrA expression in *Salmonella* enhanced its invasiveness. Liver abscesses and *Salmonella* translocation to the gallbladder were observed [52]. Further studies have emphasized the importance of AvrA in intestinal inflammation, bacterial translocation, and chronic infection associated with colon cancer in vivo [52,53,54,55].

C57BL/6 is a popular inbred mouse strain used in most laboratories. However, these mice express a functionally defective variant of *Nramp1*[56]. The *Nramp1* gene encodes an ion transporter that is expressed in macrophage and dendritic cells and is a critical host factor in defense against intracellular bacteria [57]. Thus, this variant causes C57BL/6 mice to be innately susceptible to virulent strains of Salmonella but succumb to infection in 7 days. Conversely, 129SvJ mice express a wild-type Nramp1 (Nramp1wt/wt) variant that is functionally active and as a result are capable of developing a persistent infection with virulent Salmonella strains [56]. It has been shown that if infected with virulent *Salmonella*, the bacteria is found in macrophages and lymph nodes for up to a year in *Nramp1^wt/wt^* mice or their F1 hybrids when crossed with C57BL/6 mice [58].

Not much is known regarding the mechanisms of persistent *Salmonella* infection. A negative screen isolating factor required for *Salmonella* persistence performed by Lawley and colleagues revealed that SPI-1 was necessary for sustaining persistent infection for at least 1 month post infection [59]. It is also likely that chronic infection is maintained by the re-invasion of *S*. Typhimurium into epithelial tissues after its expulsion from dying cells [60,61]. T3SS effector Sse1 is also required for long-term systemic infection in mice [62]. It is secreted by *Salmonella* from the SCV into the host cytosol and plays a role in regulating cell migration in macrophage and dendritic cells by interfering with their ability to adhere, migrate, and communicate with other parts of the immune system [62,63]. In addition, effectors ShdA and MisL, which bind to fibronectin as well as other surface structures encoded by operons *Ipf*, *bcf*, and *sth*, contribute to persistence and fecal shedding of live *Salmonella* [64,65,66,67,68,69]. In addition, effectors that are part of SPI-2 are required for chronically infected mice to transmit *S.* Typhimurium to non-infected cage mates [66,70].

Lastly, a mechanism that host cells use to minimize microbial invasion is by producing reactive oxygen species (ROS) and reactive nitrogen species (RNS) [71]. In particular, neutrophils produce myeloperoxidase that can directly bind to the cell surface of *Salmonella* allowing for targeted killing of the organism via ROS [72]. This mechanism controls the growth of *Salmonella* at sites of inflammation, but when used in conjunction with RNS, can control the overall spread of infection [73]. However, studies have indicated that mice lacking the ability to produce nitric oxide synthase succumb to *Salmonella* infection and are not chronically infected [74,75]. However, chronic inflammation can occur in vivo due to T3SS genes *sodC1* and *hmp*, which code for enzymes that detoxify superoxide and nitric acid respectively [70,76,77,78].

These characteristics help *Salmonella* to survive long-term in host cells and result in a persistent or chronic infection. Patients with chronic *Salmonella* infections do not display symptoms, but can infect other people because the bacteria can persist in the stool [79].

## 5. *Salmonella* Infection and Chronic Inflammatory Diseases

The intestinal mucosa contains more than 80% of activated B cells, and the intestine is the main antibody-producing tissue of the body [80]. Thus, infections in the intestine often cause gastrointestinal disorders and impaired immune functions, all of which result in intestinal or systemic autoimmune diseases like IBD (inflammatory bowel disease) [4] and arthritis [2,3]. The pathogenesis of IBD involves the following three aspects: (1) impaired intestinal mucosal barrier function, (2) gut microbiota changes, and (3) immune regulation disorder [81].

*Salmonella* must compete with normal gut microbiota for nutrients and intestinal epithelial attachment sites. This competitive relationship is known as colonization resistance of gut microbiota. Some studies have shown that *Salmonella* can alter colonization resistance in the intestine of mice, destroy normal gut microbiota, and cause colitis [82,83]. *Salmonella* infection increases susceptibility to intestinal inflammation and contributes to IBD [84]. However, the disorder of the host immune system caused by infection is the key factor for *Salmonella*-induced IBD. When the intestinal mucosal barrier is destroyed upon *Salmonella* infection, pro-inflammatory pathways are activated. The binding of TLRs and NOD proteins to bacterial surface moieties and toxins activates the NF-κB pathway leading to the production of cytokines and chemokines. The basolateral secretion of IL-8 recruits neutrophils and granulocytes into the subepithelial layer. Macrophages are also activated through their TLRs in the subepithelial region and induce the production of IL-1, IL-6, and IL-23, all of which drive the differentiation of Th17 cells which further induce an inflammatory state, recruiting and activating neutrophils to clear out the bacterial infection. In addition, IL-8 and IL-12 secreted from activated macrophages drive IFN-γ-dependent production of antigen specific Th1 cells [20,22,85].

In addition to promoting bacterial entry, many effector proteins encoded by SPI-1 activate inflammatory signaling. The activation of Rho family GTPases by SopE and SopB results in the activation of MAPK, ERK, JNK, and p38 signaling and subsequent induction of NF-κB encouraging a proinflammatory state [22,86]. *Salmonella* effector protein AvrA stabilizes the expression of tight junction proteins (e.g., ZO-1) and plays a role in reducing inflammation in *Salmonella*-induced colitis in in vivo models [39,40]. AvrA can inhibit epithelial cell apoptosis by regulating c-Jun N-terminal kinase to reduce epithelial cell damage [78]. In addition, *Salmonella* targets the tight junction protein claudin-2 to facilitate bacterial invasion by activating the EGFR pathway [87]. The *S.* Typhimurium *lpf*, *bcf*, *stb*, *stc*, *std*, and *sth* fimbriae operons are known to be required for intestinal persistence in mice [33]. The balance between protection and injury could be an underlying mechanism of *Salmonella* chronic infection.

Chronic *Salmonella* infection also leads to intestinal fibrosis. Mouse studies have shown that fibrosis appears at day 7, peaking at day 21, and persists to day 70 after *Salmonella* infection [88]. *Salmonella*-induced fibrosis in mice is a practical way to build an animal model needed to study intestinal fibrosis [89,90]. The underlying mechanism of intestinal fibrosis caused by *Salmonella* is linked to MyD88 and Cox-2 signaling. MyD88 (−/−) mice produce less collagen and have fewer fibroblasts in the submucosa compared with WT mice. The Cox-2 inhibitor rofecoxib could reverse the process of fibrogenesis in WT mice [91].

## 6. *Salmonella* Infection Increases Risk of Gallbladder Cancer and Colon Cancer

The link between viral infections and their roles in inducing cancers such as cervical and liver cancer has been confirmed, but the role in which bacteria contributes to tumorigenesis is poorly understood. The most widely accepted example of this is that *Helicobacter pylori* infections can contribute to gastric cancer [92]. Chronic inflammation of the gastric mucosa by *Helicobacter pylori* can activate the Wnt/β-catenin pathway stimulating carcinogenesis [93]. Research has shown that chronic *S.* Typhoid and *S.* Paratyphoid carriers have higher risk for cancer of the gallbladder, pancreas, colon and lung [94]. In subsequent studies, *Salmonella* was verified as a pathogen that can induce both gallbladder [5] and colon cancer [6]. Gallbladder cancer is the sixth most common gastrointestinal cancer and the annual incidence of gallbladder cancer is about 2 per 100,000 worldwide [95,96]. There are several common genes mutated in gallbladder cancer, such as *KRAS* [97], *TP53* [98], and *C-ERB-B2* [99].

*Salmonella* Typhi infection not only causes chronic inflammation and damage of the mucosa, but also produces toxins such as cytolethal distending toxins (CDT), which contribute to DNA damage and induce cell-cycle arrest [100]. CDTs are heterotrimeric toxins comprised of CdtA, CdtB, and CdtC subunits. The CdtB subunit can activate DNAse I activity, while CdtA and CdtC contribute to the binding of the holotoxin to the plasma membrane of host cells [101]. CdtB activity relies on the expression of two genes, pertussis-like toxin A (*pltA*) and pertussis-like toxin B (*pltB*), which can form a CdtB–PltA–PltB tripartite complex that induces DNA damage and cell-cycle arrest, thereby inducing carcinogenesis in the gallbladder [102,103]. It has been shown that both *S*. Typhi and non-typhoidal *Salmonella* both contribute to gallbladder carcinogenesis. Reports have indicated that TP53 mutations and *Salmonella* chronic infection models in gallbladder cancer are analogous to the pathogenic role of *Helicobacter pylori* in gastric cancer [103]. Butin-Israeli et al stated that neutrophil-derived miR-23a and miR-155 downregulate LB1 and RAD51 leading to increased inflammation and genomic instability [104]. RAD51 plays a key role in DNA homologous recombination and can be downregulated by p53 [105,106]. In our previous study, we found that *Salmonella* Typhimurium infection increases p53 acetylation via AvrA in intestinal epithelial cells. P53 acetylation was able to induce cell cycle arrest at G0/G1 and decrease inflammation. Though AvrA increases p53 acetylation, total p53 levels were reduced [107]. We assume that decreased p53 leads to upregulation of RAD51, thereby promoting the DNA damage repair process. This process may confer a survival advantage to infected cells by increasing the activation of the DNA repair system allowing the cells to eliminate DNA mutations.

Recently, a report indicated that severe *Salmonella* infection contributes to increased risk of human colon cancer with tumors developing mainly in the ascending colon and the transverse colon [6]. *Salmonella* chronic infection and subsequent immune disorders are potential risks of colon cancer due to increased risk of salmonellosis in colon cancer patients with IBD [6]. IBD is an independent risk factor of colon cancer with about 20% of IBD patients developing colon cancer in 30 years [108]. *Salmonella* infection is a potential factor contributing to the transformation of host cells from a colitic to cancerous state through the activation of its T3SS effectors. AvrA’s deubiquitinase activity can block degradation of IκBα and β-catenin and suppress the NF-κB pathway, thereby inhibiting the host inflammatory responses [34]. It is well known that the NF-κB and Wnt/β-catenin pathways are closely related to the occurrence and development of cancer [109,110]. The expression of AvrA is reported to be higher in cancer adjacent tissues than in normal human colon and cancer tissues [53]. Therefore, *Salmonella* AvrA may lead to colon cancer by regulating the NF-κB pathway and Wnt/β-catenin. In fact, a further study showed that AvrA activates the Wnt/β-catenin signaling pathway by preventing β-catenin ubiquitination and degradation, resulting in the upregulation of Myc and cyclin D1 expression and the promotion of colon tumorigenesis [54]. The STAT3 signaling pathway is a key player both in IBD and colitis-associated cancer [111] as it can inhibit inflammation and promote proliferation. Furthermore, we have found that AvrA can activate the STAT3 signaling pathway by upregulating IL-6 [112]. Thereby, secretion of AvrA by *Salmonella* can contribute to colitis-associated cancer by upregulating STAT3, NF-κB, and Wnt/β-catenin signaling pathways.

Other bacterial effectors could also play a role in carcinogenesis. As previously stated, SopB is a T3SS effector that induces sustained activation of Akt/protein kinase B, a pro-survival kinas; activates the Akt signaling pathway; and subsequently protects epithelial cells from apoptosis [113]. Based on this report, the anti-apoptotic role of SopB could be an underlying mechanism of *Salmonella*-associated colon cancer.

## 7. Mechanisms: Transformation, Stem Cells, Microbiota

*Salmonella* infection plays an important role in cellular phenomena, such as host cell transformation, stem cell growth, inflammation, and gut microbiota regulation. *Salmonella* can induce cellular transformation by activating the Wnt/β-catenin signaling pathway. Studies report that *Salmonella* can induce follicular-associated epithelial cell transformation into M cells by epithelial-mesenchymal transition (EMT), thereby promoting its colonization and invasion [114]. GSK3β is a key complex in Wnt/β-catenin signaling, which interacts with β-catenin, causing its phosphorylation and degradation. SopB can inhibit GSK3 by activating Akt, [113] which phosphorylates β-catenin increasing its transcriptional activity and ultimately activating the Wnt/β-catenin pathway [115]. Wnt/β-catenin activation increases the expression of EMT transcriptional activators (snail, slug, and twist) and down-regulates the expression of the tumor suppressor E-cadherin [116]. T3SS is also important for neoplastic transformation of epithelial cells. It has been shown that Apc+/min mice (with c-Myc overexpression) [117] infected with WT *S.* Typhimurium developed cancer in their colon, but Apc^+^/min mice infected with ΔprgH mutant *Salmonella* (lacking functional T3SS) did not [118]. Mouse embryonic fibroblasts (MEFs) with genetic susceptibility can transform after WT *Salmonella* infection because effectors of the T3SS increase AKT and MAP kinase activities, whereas mutant *Salmonella* lacking effectors SopB, SopE, SopE2, and SptP cannot activate the AKT and MAP kinase [119]. The transformation to cancerous cells persists even after *Salmonella* has been eradicated from the host. Studies have also shown that during *Salmonella* infection, the organism can modify the transcriptome of the host cell and induce epigenetic changes [118]. In our previous studies, we demonstrated that *Salmonella* protein AvrA activates the Wnt/β-catenin pathway, which is essential for the maintenance of the intestinal stem cell niche [43]. *Salmonella* infection can also regulate the intestinal stem cell markers, Lgr5 and Bmi1 [120].

The gut microbiota is a stable and closed system because of its colonization resistance. In most cases, foreign microbes are unable to colonize a healthy gut and disrupt the gut microbiota [121]. *Salmonella* is capable of disrupting the gut flora but once the pathogen has been cleared from the gut, the microbiota population can revert back to normal [82]. *Salmonella* can colonize the gut microbiota through multiple mechanisms. Upon infection, *Salmonella* can stimulate the C-type lectin RegIIIβ which can destroy indigenous bacteria, such as *E. coli* and *Yersinia* spp., resulting in dysbiosis [122,123]. *Salmonella* can utilize ethanolamine as a nutrient to proliferate and colonize via tetrathionate respiration inside of the inflamed intestine [124]. *Salmonella* can also compete with indigenous microbiota for intestinal epithelial attachment sites and nutrients thereby disrupting gut microbiota.

**Figure 1:**
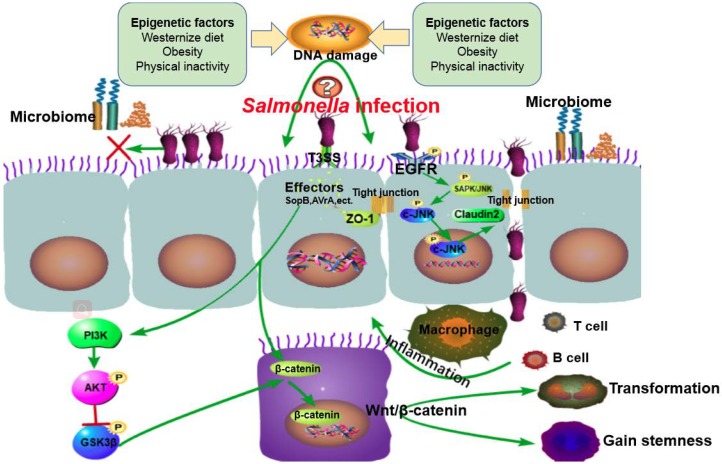
The working model summarizing the roles of Salmonella infection in cancer. Salmonella infection induces carcinogenesis potentially through four paths: (1) the host is preconditioned by DNA damage caused by epigenetic factors (Western diet, obesity and physical inactivity) and genetic factors (tumor susceptibility); (2) Salmonella effector protein AvrA stabilizes the expression of tight junction proteins (e.g., ZO-1) and decreases inflammation. Meanwhile, Salmonella targets the “leaky protein” claudin-2 to facilitate bacterial invasion. The balance between protection and injury contributes to chronic infection and leads to sustained epithelial cell injury and DNA mutation; (3) Salmonella competes with indigenous microbiota for intestinal epithelial attachment sites and nutrients, thereby disrupting the gut microbiome and overcoming colonization resistance; and (4) T3SS effectors enter epithelial cells and activates signaling pathways leading to chronic inflammation, host cell transformation, and carcinogenesis.

In summary, *Salmonella* infection may induce carcinogenesis through manipulating multiple factors (Figure 1). *Salmonella* can cause persistent intestinal infection, gut microbiota imbalance and chronic inflammation, which further induces DNA damage resulting in chromosome instability or epigenetic modification. Cancer-related signaling pathways are activated by *Salmonella* effector proteins. During chronic infection, *Salmonella* activates the Wnt/β-catenin signaling pathway, leading to host cell transformation. Leaky gut, microbiota imbalance, and inflammation are induced by bacterial proteins and contribute to the development of cancer.

## 8. Conclusion and Future Directions

*Salmonella* is a leading bacterial cause of acute gastroenteritis and constitutes a huge health burden in both developing and developed countries. Symptoms of acute infection (e.g., diarrhea, fever, abdominal pain, nausea, and vomiting) are easy to identify and treat using antibiotics or through natural clearance of the organism. However, *Salmonella* infection can exist in host cells persistently causing patients to chronically carry the pathogen resulting in further spread of the bacteria. Persistent infection can also lead to the development of other severe diseases such as IBD and cancer. Lastly, chronic *Salmonella* infection plays a role in a number of biological processes, such as stem cell maintenance, host cell transformation, and gut dysbiosis. The mechanism by which *Salmonella* leads to colitis-associated colon cancer include (1) impaired intestinal mucosal barrier function by acute infection; (2) the effectors of the T3SS activating essential host cell pathways causing immune regulation disorders and finally; (3) *Salmonella*-associated dysbiosis. *Salmonella* can act as an extremely pathogenic organism and cause extensive damage to host cells contributing to the onset of severe diseases.

*Salmonella* has benign properties that can be used to diagnose or treat cancer. It has been reported that live attenuated bacteria can destroy cancer cells in vitro and in vivo [125,126]. Specifically, recombinant attenuated *Salmonella* vaccines (RASV) have been developed to treat neuroblastoma [127]. *Salmonella* can be used as a vector to deliver chemotherapeutic drugs to cancer cells and eradicate them with precision. However, much research is needed before these practices can be used as sufficient cancer therapies as the safety of this treatment strategy has yet to be evaluated.

*Salmonella*-associated cancer involves the interplay of multiple elements, such as environmental factors, host genetic background, the gut microbiome, and host cell immunity. It should be noted that *Salmonella* infection and its chronic effects are not the same as chemically-induced biological events. *Salmonella* infection may not act in a dose-dependent manner. In the event a small number of bacteria managed to survive in the host, the opportunity could still arise for the organism to multiply and translocate to other organs. Studies using human samples and mouse models will help us further understand the relationship between *Salmonella* infections and the risk of colon cancer.
